# Bilateral Multifocal Ovarian Teratomas and Ovarian Torsion in a Young Woman With Polycystic Ovarian Syndrome: A Case Report

**DOI:** 10.1155/crog/6856494

**Published:** 2026-09-06

**Authors:** Megan R. Messina, Marcia H. Varella, Francisco J. Fajardo, Robert Hunter, Nicole Henry

**Affiliations:** ^1^ Department of Medical Education, Florida International University, Herbert Wertheim College of Medicine (FIU-HWCOM), Miami, Florida, USA, fiu.edu; ^2^ Department of Obstetrics and Gynecology, Memorial Healthcare System, Pembroke Pines, Florida, USA, mhs.net

## Abstract

**Background:**

Ovarian dermoid cysts are common benign neoplasms that typically manifest as a solitary lesion. Bilateral multifocal presentations, particularly in the context of polycystic ovarian syndrome (PCOS), are exceptionally rare and pose significant diagnostic challenges.

**Case Presentation:**

A 22‐year‐old nulligravid Caucasian woman with a history of PCOS presented with acute, severe lower abdominal pain accompanied by nausea and vomiting. Initial imaging revealed large bilateral ovarian masses with features consistent with dermoid cysts and clinical findings suggestive of a right ovarian torsion. Despite a prior computed tomography (CT) scan indicating bilateral ovarian cysts, the patient attributed her symptoms to PCOS‐related changes, delaying timely evaluation. Intraoperative findings during an exploratory laparotomy confirmed the presence of more than five distinct dermoid teratomas on each ovary. Bilateral ovarian cystectomies were performed with careful preservation of ovarian stromal tissue, and histopathological analysis verified the diagnosis of mature cystic teratomas.

**Conclusion:**

This case underscores the critical need for early differentiation between PCOS‐related cysts and ovarian dermoid cysts to avert complications such as ovarian torsion and to preserve fertility. It also highlights the essential role of clear and effective communication between healthcare providers and patients in ensuring timely diagnosis and intervention. Misinterpretation of clinical symptoms can lead to delayed presentation with potentially deleterious effects on future fertility.

**Teaching Point:**

A CT scan obtained during the patient′s initial evaluation revealed ovarian teratomas; however, the patient mistakenly believed these masses represented her typical PCOS‐associated cysts rather than pathology of a different etiology. This misconception delayed recognition of the severity of her condition and underscores the importance of clear physician–patient communication to ensure patients understand the significance of their diagnoses, particularly when fertility and overall well‐being may be affected.

**Timeline**

The timeframe of events are shown as follows:

• Age 17: diagnosis of PCOS.
• 7 months prior: intermittent mild abdominal pain begins.
• 5 months prior: CT scan shows bilateral ovarian cysts.
• 2 days prior to ED visit: severe right lower quadrant pain with nausea/vomiting.
• Day of admission: ED presentation; imaging confirms dermoid cysts.
• Hospital course: exploratory laparotomy with detorsion and bilateral cystectomies.
• Postoperative Day 3: discharged home in stable condition.

## 1. Background

A dermoid cyst is a mature cystic teratoma arising from totipotent embryonic germ cells, most commonly found in the ovaries. The majority of these cases are diagnosed in childhood or early adulthood. Dermoid cysts represent the most common benign ovarian neoplasm in women between the ages of 20 and 40 years, accounting for approximately 10%–15% of all ovarian tumors [1]. Ovarian dermoid cysts grow slowly and are mostly asymptomatic, unilocular, less than 5 cm in diameter, and typically unilateral [1]. A 2005 study of 270 histologically and sonographically confirmed ovarian cystic teratomas reported found that 73% were right‐sided, 17.4% left‐sided, and only 10.37% bilateral [2]. Despite being benign, their size and location can lead to complications. Most often, dermoid cysts remain asymptomatic and can be monitored through periodic imaging; however, symptomatic or enlarging cysts are best managed with surgical excision. Large dermoid cysts can lead to torsion, ischemia, necrosis, rupture, infection, and potential fertility loss, making prompt intervention essential to preserve ovarian stromal viability. Overall, complete cyst excision generally results in an excellent prognosis with a low risk of recurrence or malignant transformation.

Multiple dermoid cysts in the same ovary are exceedingly rare, and even fewer cases exist in the literature that are reported as multiple and bilateral [3–7]. We report a case of a 22‐year‐old female with this rare presentation of bilateral polydermoid ovaries.

The rarity of this condition underscores the extraordinary nature of the clinical finding, highlighting challenges and implications in distinguishing between PCOS‐related cysts and neoplastic dermoid cysts, and emphasizing the need for clear communication between healthcare providers and patients. Ensuring that patients fully understand their diagnosis and the potential risks, such as ovarian torsion and fertility loss, is critical for facilitating timely and informed decision‐making. This case highlights how pre‐existing gynecologic diagnoses such as PCOS may influence patient interpretation of new imaging findings, emphasizing the importance of thorough counseling regarding differential diagnoses and associated risks.

## 2. Case Report

A 22‐year‐old nulligravida Caucasian woman presented to the emergency department (ED) with acute‐onset lower abdominal pain. The woman had a history of polycystic ovarian syndrome (PCOS) diagnosed at 17 years of age. In the last 7 months, she experienced occasional, intermittent, mild, crampy abdominal pain localized to the lower abdomen. Computed Tomography (CT) scan 5 months prior to this presentation revealed large bilateral ovarian cysts. Despite progressive worsening of her symptoms, the patient did not follow‐up with a gynecologist as she believed the cysts were due to her known diagnosis of PCOS.

For 2 days prior to her ED visit, the patient experienced constant, sharp, severe pain in the right lower quadrant, which was worse with movement and unrelieved by Ibuprofen. She also vomited 10 times over a 24‐h time frame.

### 2.1. Past Medical/Surgical History

She was PCOS‐diagnosed at 17 and treated with metformin from 17 to 21. Surgical history was noncontributory.

### 2.2. Gynecological History

Her gynecological history includes menarche at 13 and irregular cycles that were managed with oral contraceptives from ages 14 to 16. She currently has 28‐day cycles, a normal Pap smear from 1 year ago, no history of STDs, and is not currently sexually active.

### 2.3. Family History

Her maternal grandmother had unilateral breast cancer (died at 40); mother had skin cancer in her 50s with no breast or gynecologic malignancies; and paternal grandfather had hypertension and diabetes (died in his 70s). She had no known genetic conditions.

### 2.4. Social History

The patient denies the use drugs, alcohol, and tobacco.

### 2.5. During Admission

On physical examination, the patient was in acute distress and writhing in pain. Her vitals include the following: temp. 36.6°C, BP 142/79, HR 87, RR 17, SpO2 98%, and BMI 44.09. Abdominal examination revealed exquisite tenderness, especially at McBurney′s point and positive rebound and guarding.

### 2.6. Genitourinary Examination

Speculum exam revealed a closed external os and scant dark red blood in the vaginal vault. Moderate suprapubic tenderness was noted. The ovaries were difficult to palpate due to the patient′s large body habitus and pain.

### 2.7. Laboratory Findings

Results on admission included CBC with elevated WBC 13.5 and ANC 9.89. CMP demonstrated an elevated ALT 47 and AST, and all else was normal.

### 2.8. Imaging

**Figure 1:**
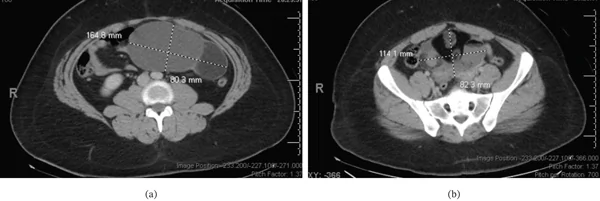
(a) Axial computed tomography of the left ovary reveals a well‐circumscribed, heterogeneous dermoid. The mass contains areas of variable attenuation, including fat densities and possible calcifications, consistent with a mature dermoid cyst. The mass exerts mild mass effect on the adjacent structures without evidence of infiltration or peritoneal free fluid. (b) Axial computed tomography of the right ovary reveals a complex, heterogeneous dermoid within the right adnexa. The mass exhibits mixed attenuation, including fat densities and possible calcifications, consistent with the characteristics of a mature cystic dermoid. The mass exerts mild mass effect on the adjacent pelvic structures with no evidence of infiltration.

Pelvic ultrasound (transabdominal, transvaginal, and Doppler) revealed an inhomogeneous uterus with a 1‐cm endometrial stripe and no discrete uterine mass. A complex right adnexal mass (11.6 cm) with fatty, cystic components and small calcifications, consistent with a dermoid cyst, was identified. The left ovary was poorly visualized. Doppler of the right ovary showed normal color flow (resistive index 0.6). Contrast‐enhanced CT of the abdomen and pelvis demonstrated large bilateral ovarian dermoids with slight growth compared with 5 months prior. The left ovary (Figure 1a) measured 16.5 × 8.0 cm (previously 12.8 × 7 cm), and the right ovary (Figure 1b) measured 11.4 × 8.2 cm (previously 12.8 × 7 cm). No bowel obstruction, thickening, or other organ abnormalities were identified.

### 2.9. Clinical Course

Ovarian torsion was suspected due to the patient′s acute‐onset of lower abdominal pain and history of bilateral ovarian cysts. CT scan showed no signs of appendicitis but revealed significant enlargement of the bilateral ovarian cysts, further supporting a diagnosis of ovarian torsion.

**Figure 2:**
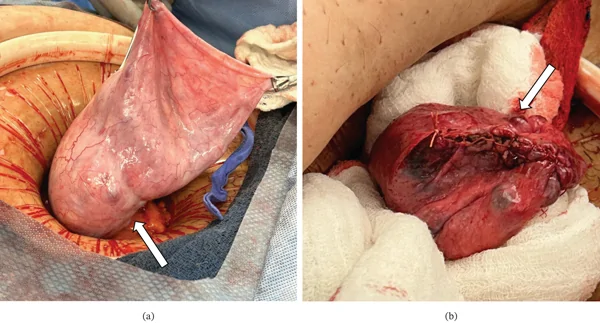
(a) Intraoperative gross pathology findings reveal a markedly enlarged left ovary that appears grossly distended and encapsulated with a smooth, glistening serosal surface. There is no obvious rupture or evidence of surface irregularity. The ovary appears to contain multiple cysts within the parenchyma (white arrow). (b) Postoperative gross examination of the left ovary after multiple cystectomies (white arrow).

**Figure 3:**
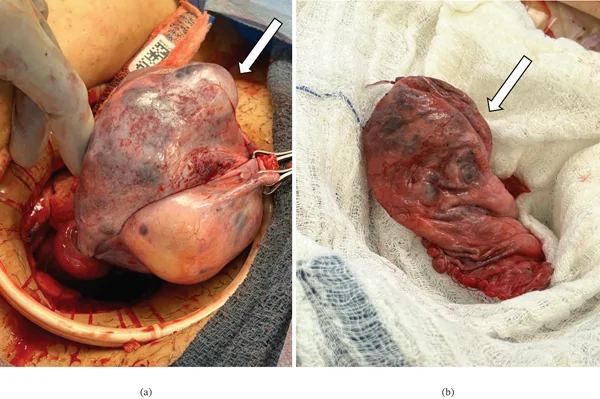
(a) Intraoperative gross examination reveals a markedly enlarged, torsed right adnexa containing multiple ovarian cysts (white arrow). (b) Right ovary following detorsion and multiple cystectomies (white arrow).

**Figure 4:**
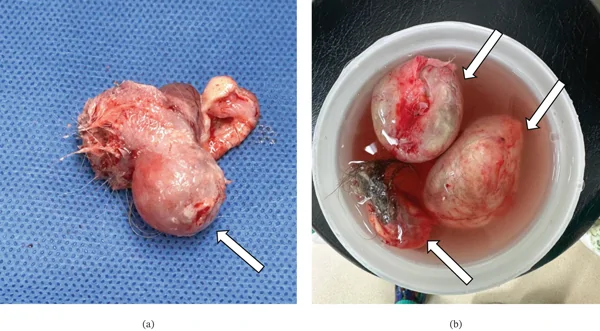
(a) Gross specimen of a surgically excised appearing dermoid cyst (white arrow). Intradermoid contents are exposed, revealing sebaceous material and hair, characteristic of a mature cystic dermoid. (b) The specimen container holds multiple excised dermoid cysts (white arrow). Intradermoid contents are visible, including sebaceous material and hair.

The patient underwent an exploratory laparotomy with reduction of the right ovarian torsion with bilateral ovarian cystectomies. The intraoperative findings included right ovarian torsion and more than five distinct cyst‐walled dermoid cysts in each ovary (Figures 2, 3, and 4). Her recovery course was uneventful and she was discharged home on postoperative Day 3.

## 3. Operative Findings

Exploratory laparotomy revealed a markedly enlarged, torsed right ovary with edema and vascular compromise. Following detorsion, improvement in ovarian perfusion and coloration was observed. Bilateral ovaries contained numerous mature cystic teratomas, with more than five distinct cyst‐walled dermoid cysts identified within each ovary. Bilateral ovarian cystectomies were successfully performed while preserving the remaining ovarian tissue. Representative intraoperative findings of the enlarged ovaries, ovarian torsion, and bilateral dermoid cysts are shown in Figures 2a,b, 3a,b, and 4a,b. Gross examination of the excised specimens demonstrated characteristic mature cystic teratomas containing sebaceous material and hair (Figure 4a,b). Hemostasis was achieved, and the abdomen was closed in the standard fashion.

## 4. Discussion

We present a case of a young woman with rare bilateral polycystic dermoid teratomas and PCOS. Dermoid cysts are diagnosed based on clinical presentation, imaging, and histopathological examination. Clinically, they may present as a nontender mobile mass or with acute pain if torsion or rupture happens. Ultrasound, MRI, and CT scans are crucial for the diagnosis. Ultrasound reveals the characteristic mixed echogenicity. MRI and CT being useful to identify deeper lesions. Histopathology ultimately distinguished the mass as a benign mature cystic teratoma, demonstrating characteristic benign features including well‐differentiated ectodermal elements such as squamous epithelium, sebaceous material, and hair follicles without evidence of immature tissue, atypia, or malignant transformation. In this particular case, the patient′s previous PCOS diagnosis likely delayed her from seeking care as she attributed her pain to this pre‐existing condition. Although the rarity of bilateral polydermoid ovaries confused the clinical picture, clear and timely communication between provider and patient could have allowed for more prompt diagnosis and care. Early differentiation is vital to prevent complications like ovarian torsion and to preserve fertility in reproductive‐aged women. Prompt surgical intervention was crucial in young women to preserve ovarian function, with the approach tailored to minimize damage to healthy ovarian tissue.

## Author Contributions

Megan R. Messina: conceptualization (lead), writing—original draft (lead), writing—review and editing (equal), visualization (lead), resources (supporting), and project administration (lead). Nicole Henry: conceptualization (lead), data curation (lead), supervision (lead), and writing—review and editing (equal). Marcia H. Varella: conceptualization (lead) and writing—review and editing (equal). Francisco J. Fajardo: resources (lead) and writing—review and editing (equal). Robert Hunter: supervision (supporting) and writing—review and editing (equal).

## Funding

No funding was received for this manuscript.

## Ethics Statement

This study was conducted in accordance with institutional ethical standards. Ethical approval was not required for this case report in accordance with local institutional policies. Written informed consent was obtained from the patient for publication of this case report and accompanying images.

## Conflicts of Interest

The authors declare no conflicts of interest.

## Data Availability

Data sharing is not applicable to this article as no datasets were generated or analyzed during the current study.
